# Serum proteomic profiles of depressive subtypes

**DOI:** 10.1038/tp.2016.115

**Published:** 2016-07-12

**Authors:** F Lamers, M Bot, R Jansen, M K Chan, J D Cooper, S Bahn, B W J H Penninx

**Affiliations:** 1 Department of Psychiatry and EMGO Institute for Health and Care Research, VU University Medical Center, Amsterdam, The Netherlands; 2 Department of Chemical Engineering and Biotechnology, Institute of Biotechnology, University of Cambridge, Cambridge, UK

## Abstract

Depression is a highly heterogeneous disorder. Accumulating evidence suggests biological and genetic differences between subtypes of depression that are homogeneous in symptom presentation. We aimed to evaluate differences in serum protein profiles between persons with atypical and melancholic depressive subtypes, and compare these profiles with serum protein levels of healthy controls. We used the baseline data from the Netherlands Study of Depression and Anxiety on 414 controls, 231 persons with a melancholic depressive subtype and 128 persons with an atypical depressive subtype for whom the proteomic data were available. Depressive subtypes were previously established using a data-driven analysis, and 171 serum proteins were measured on a multi-analyte profiling platform. Linear regression models were adjusted for several covariates and corrected for multiple testing using false discovery rate *q*-values. We observed differences in analytes between the atypical and melancholic subtypes (9 analytes, *q*<0.05) and between atypical depression and controls (23 analytes, *q*<0.05). Eight of the nine markers differing between the atypical and melancholic subtype overlapped with markers from the comparison between atypical subtype and controls (mesothelin, leptin, IGFBP1, IGFBP2, FABPa, insulin, C3 and B2M), and were mainly involved in cellular communication and signal transduction, and immune response. No markers differed significantly between the melancholic subtype and controls. To conclude, although some uncertainties exist in our results as a result of missing data imputation and lack of proteomic replication samples, many of the identified analytes are inflammatory or metabolic markers, which supports the notion of atypical depression as a syndrome characterized by metabolic disturbances and inflammation, and underline the importance and relevance of subtypes of depression in biological and genetic research, and potentially in the treatment of depression.

## Introduction

Despite major efforts to unravel the pathophysiological mechanisms of major depressive disorder (MDD) through the analysis of genome-wide association study data^1^ and RNA gene expression studies,^2, 3^ little insight has been gained and it was only recently that a first genetic hit for MDD was found in a female sample of cases of recurrent depression.^4^ With the availability of new proteomic techniques that allow for the simultaneous quantitative measurement of a wide array of proteins, it is now possible to study the actual functional profiles of proteins, protein–protein interaction and pathways. Proteins are the end products of RNA and DNA, and often the functional and modifiable units in disease mechanisms. Consequently, a proteomic approach may provide a more complete and useful representation of pathophysiological processes potentially involved in MDD.

Several studies have applied proteomic approaches in MDD populations in the past few years^5, 6, 7, 8, 9, 10^ (2–7 years) most of them with a relatively small sample size (range *N*=12–49) and four studies with larger sample sizes^11, 12, 13, 14, 15^ (range *N*=102–1589), of which one was conducted by our group.^11^ Proteins differentially expressed in these studies were mostly involved in inflammation, insulin-related pathways and metalloproteinases.

A major factor hindering the further identification of endophenotypes, reproducible biomarkers and genes related to depression is the large heterogeneity in the presentation of depression. Consequently, an increasing number of studies now attempt to unravel pathophysiological mechanisms by distinguishing more homogeneous depressive subtypes. Two previous proteomics studies have distinguished between MDD subtypes.^6, 7^ One study found different protein profiles between MDD patients with and without psychosis^7^ and the other study differentiated between MDD with and without childhood trauma.^6^ Two other relevant subtypes, suggested in a comprehensive overview of subtype differences by Gold and Chrousos,^16^ include melancholic and atypical depression. Recent studies that distinguished atypical depression and melancholic depression using either DSM-IV criteria or data-driven classifiers, have shown that especially atypical depression is associated with obesity, metabolic syndrome and inflammation, whereas it is melancholic depression that is most strongly associated with hyperactivity of the HPA-axis.^17, 18, 19, 20^

To our knowledge, no study has investigated whether atypical and melancholic subtypes have different molecular signatures using a proteomic approach, and we therefore set out to evaluate this using the data from the Netherlands Study of Depression and Anxiety (NESDA). A previous analysis of all current (diagnosis in past 6 months) and remitted cases of MDD and healthy controls from the NESDA sample^11^ suggested associations of 33 analytes with MDD, of which seven analytes (out of 16 of the 33 available) were also associated with MDD in validation cohorts. Application of proteomic approaches to differentiate atypical and melancholic depression could increase our understanding of subtype-specific pathophysiological processes. The aim of the current study was therefore to evaluate differences in serum protein profiles between persons with atypical and melancholic depressive subtypes, and to compare these profiles with serum protein levels of controls.

## Materials and methods

### Sample

NESDA is an ongoing naturalistic longitudinal cohort study (*N*=2981) of persons with depressive and/or anxiety disorders and healthy controls, recruited from the community, primary care and secondary care.^21^ The study was approved by the Ethical Review Board of the VU University Medical Centre and subsequently by local review boards of each participating centre. At baseline, participants visited the study site for an extensive assessment including a psychiatric diagnostic interview, medical assessment, blood draw and self-reported questionnaires. Proteomic analytes were determined in the subset of NESDA participants who participated in both baseline and 2-year follow-up assessments and for whom sufficient serum (~1 ml) from the baseline assessment was available (*n*=1837).^11^ In the current analysis, we included persons with proteomic data for whom we had previously established as either an atypical (*n*=128) or melancholic (*n*=231) depressive subtype^11, 22^ (see below), and controls without lifetime depressive and/or anxiety disorder according to the CIDI diagnostic interview (*n*=414), giving a total sample of 773 persons.

### Assessment of depressive subtypes

In all participants, the presence of depression was determined by the CIDI diagnostic interview (version 2.1),^23^ administered by specially trained staff, using DSM-IV algorithms. The depressive subtypes of the current study were previously determined in 818 NESDA participants within a 1-month diagnosis of MDD (*n*=743) or minor depression (*n*=75) using the baseline data.^11, 22^ In short, depression items from the CIDI diagnostic interview and a selection of items from the Inventory of Depressive Symptomatology-self report (IDS-SR_30_)^24^ were input variables for a latent class analysis, which was used to cluster persons with similar symptom profiles. A three-class model was found to fit the data best. On the basis of symptom probabilities, the subtypes were labeled as ‘severe melancholic’ (prevalence 46.3%), characterized mainly by decreased appetite and weight loss. This subtype also had the highest probabilities of suicidal thoughts, psychomotor changes and lack of responsiveness. The second subtype was labeled as ‘severe atypical’ (24.6%). This severe atypical subtype was mainly characterized by overeating and weight gain, and had the highest probabilities of leaden paralysis and interpersonal sensitivity. The stability over time of both the severe melancholic and the severe atypical depression subtypes was found to be high.^25^ The last subtype was labeled as ‘moderate’ (29.1%) and was associated with lower symptom probabilities and overall lower severity. Mean posterior probabilities were 0.80 for the melancholic class, 0.88 for the atypical class and 0.84 for the moderate class showing acceptable qualification quality. In the current study, we included only the severe melancholic and the severe atypical subtypes as they are both recognized in the literature and DSM-5. Importantly, these subtypes were of a similar severity, which reduces the risk of any observed effects resulting from severity differences.

It should be noted that our latent class analysis-based subtypes of melancholic and atypical depression do not literally resemble DSM classification, in the sense that mood reactivity in atypical depression was not a cardinal item and that the number of subtype-specific symptoms does not follow DSM classification. However, the robustness of the identified subtypes is shown by other latent modeling studies finding similar symptom patterns with appetite and weight being the most discriminating symptoms.^26, 27, 28, 29, 30^ In this paper, we will use the terms ‘atypical’ and ‘melancholic’ when referring to our latent class analysis-based subtypes. A more thorough description of subtypes and their correlates has been previously published.^22, 31^

### Proteomics

Blood samples were taken after an overnight fast and stored at −80 °C in five different labs throughout the Netherlands, using standardized protocols. A panel of 243 analytes involved in various hormonal, immunological, metabolic and neurotrophic pathways were assessed in serum using multiplexed immunoassays in a Clinical Laboratory Improvement Amendments (CLIA)-certified laboratory (Myriad Rules Based Medicine; Austin, TX, USA; DiscoveryMap 250+). Of the 243 analytes assessed, 171 analytes could be included in the analysis (see Statistics section). A full list of the 243 analytes can be found in Supplementary Table 1. Six quality controls (with low, medium and high concentrations of the analyte) were included on each plate. Mean inter-assay variability was 10.6% (range 5.5–32.5%) and mean intra-assay variability was 5.6% (range 2.5–15.8%). A more detailed description can be found elsewhere.^11^

### Covariates

Sex and age information was collected using standard questions. Lab was also included as covariate. Weekly alcohol intake was derived from the Alcohol Use Disorders Identification Test (AUDIT).^32^ Smoking was defined as current smoking (Y/N). Cardiovascular disease, including cerebrovascular disease, myocardial infarction, angina pectoris and coronary heart disease, was based on self-report information or use of beta blocking agents, calcium channel blocking agents or nitrate vasodilators (ATC codes: C07, C08 and C01DA). Diabetes mellitus (DM) was based on medication use (ATC codes: A10) or fasting glucose levels ⩾7 mmol l^−1^.

In addition, the following covariates were considered for sensitivity analyses: any anxiety disorder (1-month CIDI diagnosis of social phobia, panic disorder, agoraphobia, or generalized anxiety disorder), antidepressant use including selective serotonin reuptake inhibitors (SSRIs; ATC code N06AB), tricyclic antidepressants (TCAs; ATC codes N06AA) and other antidepressants (ATC codes N06AF, N06AG, N06AX), corticosteroid use (ATC codes H02, R03BA, R03AK, D07) and anti-inflammatory medication use (ATC codes M01A, M01B, A07EB, A07EC), and body mass index (BMI; weight in kg/m^2^).

### Statistics

Of the 243 analytes assessed, 171 analytes with <30% missing values (mostly due to levels outside the limits of detection) were included in the analysis. Missing values that were below and above the limits of detection were imputed with the values of the lower and upper limit of detection, respectively. Other missing values (one missing on average per analyte) were imputed by the median value of the analyte. These imputation methods are similar to methods used in other studies.^12, 33^ Data were then log10-transformed to stabilize the variance distributions. The percentage of missing values for each analyte is listed in Supplementary Table 1. We applied the ComBat procedure^34^ to the proteomic data to remove any potential plate effects prior to analysis.

Baseline characteristics were compared using *χ*^2^-tests, analysis of variance and Kruskal–Wallis tests, where appropriate. Separate linear models were run with each analyte as the dependent variable. Besides a group variable (melancholic subtype, atypical subtype or control), models contained the following covariates: age; sex; lab; smoking; alcohol intake; cardiovascular disease; and diabetes. Comparisons between subtypes, and between subtypes and controls were performed. To account for multiple testing, a false discovery rate (FDR) was calculated.^35^ All analyses were conducted in R software (version 3.0.2)^36^ and two-sided tests were used.

To evaluate the robustness of findings, we performed additional analyses by running three models additionally corrected for potential confounding variables. In one model, we added antidepressant use, in the second we added use of anti-inflammatory agents and corticosteroids, and in the third model we added comorbid anxiety to the standard set of covariates (as listed above). To evaluate the potential mediating effect of BMI, we also repeated the analyses with BMI as covariate. Only analytes that were significant (FDR-controlled *q*<0.05) in at least one of the comparisons from the main analysis were included in these additional models. We did not apply FDR correction in these additionally corrected models.

To aid interpretation of findings, we looked up the biological processes in the Human Protein Reference Database by uploading SwissProt accession numbers of proteins. We tested for statistical overrepresentation of biological processes using PANTHER tools,^37, 38^ using the 171 tested markers as reference set and applying a Bonferroni correction. Finally, a literature search was performed in PubMed to describe the identified markers and their previously observed associations with major depression, MDD subtypes or depressive symptoms. We structured our narrative review of the literature for each of the main biological processes.

## Results

### Sample description

Persons with atypical and melancholic depression were slightly older, had more chronic diseases, and were more often using medication than controls (Table 1). Persons with atypical depression were more often female, had a higher BMI and a lower alcohol intake than controls and persons with melancholic depression. Persons with melancholic depression were most often smokers compared with the other two groups. Severity of depression was not different between melancholic and atypical subtypes (group comparison *P*=0.59).

### Differences between atypical depression, melancholic depression and controls

From the 171 analytes measured, we found 9 analytes to be differentially expressed between atypical and melancholic depressive subtypes (*q*<0.05; Table 2). Compared with melancholic depression, persons with atypical depression had higher levels of leptin, angiotensin-converting enzyme (ACE), fatty-acid-binding protein–adipocyte (FABPa), complement C3, insulin and beta-2-microglobulin (B2M), while having lower levels of insulin-like growth factor-binding protein 1 (IGFBP1), IGFBP2 and mesothelin (MSLN).

**Figure 1:**
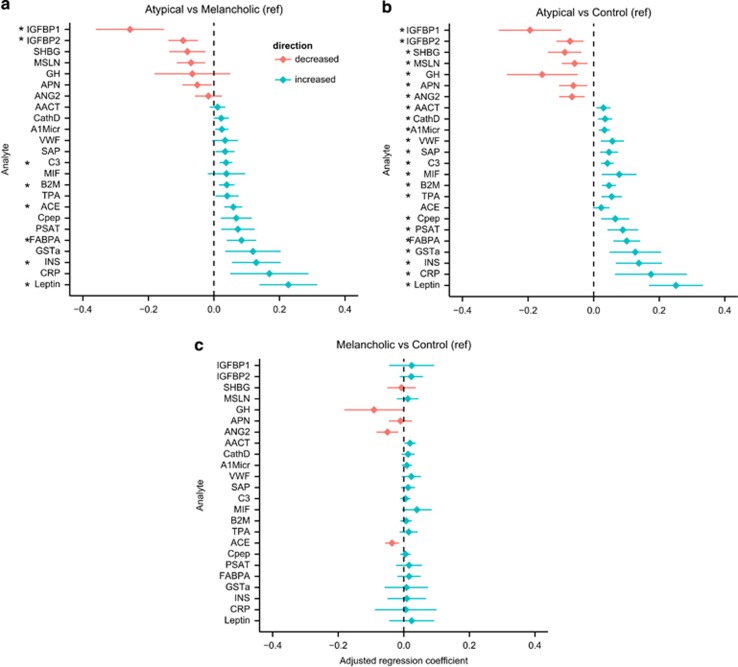
Proteins differentiating between (**a**) atypical vs melancholic depression, (**b**) between atypical depression vs controls and (**c**) between Melancholic depression vs Controls. *q<0.05, adjusted regression coefficients and error bars (s.e.). Analytes ordered based on size-adjusted regression coefficient of atypical vs melancholic depression comparison. A1Micr, alpha-1-microglobulin; AACT, alpha-1-antichymotrypsin; ACE, angiotensin-converting enzyme; ANG2, angiopoetin-2; APN, adiponectin; B2M, beta-2-microglobulin; C3, Complement C3; CathD, Cathepsin D; Cpep, C-peptide; CRP, C-reactive protein; FABPA, fatty-acid-binding protein, adipocyte; GH, human growth hormone; GSTa, glutathione *S*-transferase alpha; IGFBP1, insulin-like growth factor-binding protein 1; IGFBP2, insulin-like growth factor-binding protein 2; INS, insulin; MIF, macrophage migration inhbitory factor; MSLN, mesothelin; PSAT, phosphoserine aminotransferase; SAP, serum amyloid P-component; SHBG, sex hormone-binding globulin; TPA, tissue-type plasminogen activator; VWF, von Willebrand factor.

Except for ACE, all analytes significantly differing between atypical and melancholic subtypes also differed significantly between those with the atypical subtype and controls. The direction of effects for these eight analytes was similar to that in the comparison of atypical versus melancholic (that is, analytes being increased in atypical vs controls were also increased in atypicals vs melancholic; Figure 1; Supplementary Figure 1). In addition, 15 more analytes were significantly different between persons with atypical depression and controls. Levels of phosphoserine aminotransferase, alpha-1-microglobulin, serum amyloid P-component, tissue-type plasminogen activator, glutathione *S*-transferase alpha (GSTa), C-reactive protein (CRP), Cathepsin D (CathD), von Willebrand factor (VWF), C-peptide, macrophage migration inhibitory factor (MIF) and alpha-1-antichymotrypsin (AACT) were higher, whereas levels of adiponectin (APN), growth hormone (GH), sex hormone-binding globulin (SHBG) and angiopoietin-2 (ANG2) were lower in persons with atypical depression versus controls. Although these 15 analytes did not reach significance (*q*<0.05) in the comparison atypical versus melancholic depression, 9 of the 15 analytes (60%) had *P*-values <0.05.

There were no significant differences in levels of analytes when comparing persons with melancholic depression and controls after FDR correction. However, for three analytes (ACE, ANG2 and AACT), a *P*-value <0.05 was observed (Table 2). Supplementary Table 2 contains the regression coefficients for all group comparisons and all 171 examined analytes.

Sensitivity analyses with additional correction for antidepressant use, and corticosteroid or anti-inflammatory medication use yielded very similar results (data not shown) with all markers remaining significantly related to atypical depression (*P<*0.05) after taking these factors into account. In models with correction for comorbid anxiety, all other markers remained significant (*P<*0.05) with the exception of MSLN and ANG2 in the atypical versus control comparison (*P<*0.10). When evaluating the mediating influence of BMI, we found that significance for most markers was lost when correcting for BMI, pointing to a mediating effect of BMI. Insulin growth factor-binding protein 1, angiotensin-converting enzyme and B2M remained significant (*P<*0.05) in the comparison atypical versus melancholic depressive subtypes, whereas B2M, angiopoietin-2 and VWF remained significant in the comparison atypical depression versus control when adding BMI to the model.

### Biological processes

To identify biological processes involved in the analytes associated with MDD, we entered all 24 analytes with a significant *q*-value in any of the pairwise comparisons simultaneously in the enrichment analysis. We used the 24 analytes because we did not find differences between the melancholic subtype and controls, and because of the large overlap in analytes differing between atypical and controls and those differing between atypical and melancholic depression. We did not observe statistical overrepresentation of any biological process. The biological processes to which the largest number of analytes were linked were cell communication and signal transduction (nine analytes), protein metabolism (six analytes), immune response (five analytes), metabolism (three analytes), and energy pathways (three analytes; Table 2).

### *Post hoc* evaluation of homogeneous versus heterogeneous phenotype use

Because we suspected that ignoring heterogeneity in the symptom presentation of MDD would dilute associations with analytes, we repeated the analysis for the 171 markers, this time combining the atypical and melancholic depressive subtypes into one heterogeneous depression group and comparing them with controls, while using the same covariates as in the main analysis. We then evaluated the fold change in the estimate (calculated as estimate for the homogeneous atypical subtype versus control divided by the estimate for the heterogeneous depression (atypical+melancholic combined) versus control). The fold change in estimate ranged from −14.7 to 256.0 (median 1.45 (interquartile range 0.47–2.34)), demonstrating that associations are generally diluted when a more heterogeneous phenotype is used. Also, for 31 analytes, the direction of the effect changed. When looking at the 24 markers that were significantly different between atypical depression and controls, 9 (37.5%) did no longer reach significance at the *P<*0.05 level when comparing heterogeneous depression vs controls (that is, ACE, IGFBP2, IGFBP1, MSLN, SHBG, GSTa, CRP, CPep and APN).

### Identified analytes: roles and previous findings

An overview of previously observed associations of the analytes with depression or with other psychiatric disorders and other relevant studies is given below, ordering analytes by biological process (as listed in Table 2). For a complete overview of biological functions of the analytes, see Supplementary Table 3.

### Cell communication and signal transduction

Consistent with the present study, insulin was found to be increased in MDD in one proteomic study,^13^ but not in our previous proteomic analysis including all MDD cases in the cohort.^11^ Our finding that leptin was increased in atypical depression is consistent with a previous study in atypical depression^39^ and more recently was confirmed in the NESDA sample that also confirmed the absence of an association with melancholic depression.^40^ It implies leptin resistance—which is thought to contribute to depressive symptomatology^41^—as an underlying mechanism in atypical depression. FABPs have received attention from the depression field because fatty acid metabolism regulates neurotransmitter signaling, inflammation and thrombosis and could be a possible mechanism for the association between depression and cardiovascular disease.^42^ No differences in IGFBP levels in MDD compared with controls were found in two previous studies,^43, 44^ but one of these studies found lower IGFBP2 expression levels in brain tissue of persons with bipolar disorder compared with controls.^43^ Decreased GH levels in MDD were also previously found in two studies,^13, 14^ whereas another study found increased GH levels in elderly MDD patients only.^44^ Gold and Chrousos^16^ posed that the GH axis in both melancholic and atypical depression may be suppressed, however, we only observed this in atypical depression. MIF is a proinflammatory cytokine that has been linked to hippocampal neurogenesis in animal models.^45^ Two studies also found increased levels of MIF in depressed cases compared with controls,^14, 46^ and MIF is now increasingly gaining attention as a potential depression marker.^47^ Interestingly, increased MIF has been associated with lower morning cortisol levels,^48^ which fits in with a previous finding of lower cortisol on awakening in atypical depression compared with melancholic depression and controls.^49^ ANG2 levels were lower in atypical depression versus controls, which is in line with a previous analysis of all MDD cases within the same cohort.^11^

### Protein metabolism

One marker may possibly be considered as a melancholic depression-specific marker. ACE was significantly lower in melancholic depression than in atypical depression and also lower in melancholic depression than in controls (albeit with a non-significant *q*-value; *P*=0.0001, *q*=0.19). This finding is in line with results from a previous study on melancholic depression.^50^ Aldosterone and the renin–angiotensin system have been proposed as potential biomarker and/or mediator in the pathophysiology of MDD.^51^

AACT, an acute phase protein, has been previously found to be increased in MDD and is correlated to depression severity.^52^ Serum amyloid P-component, increased in atypical versus controls, has previously been found to be increased in MDD and schizophrenia compared with controls.^9^ Higher levels of AACT and lower levels of serum amyloid P-component have been associated with cognitive decline and with Alzheimer’s disease.^53, 54^

Two markers involved in blood coagulation processes were increased in persons with atypical depression compared with controls, VWF and tissue plasminogen activator. The VWF finding is in line with a previous analysis of all MDD cases within the same cohort,^11^ and likewise, the increased cathepsin D levels in the atypical subtype were observed before in the entire MDD group.^11^

### Immune response

As in a previous enzyme-linked immunosorbent assay of high-sensitivity CRP in this data,^49^ we found that CRP was significantly increased in atypical depression versus controls (*q*=0.02). Increased levels of complement C3 in MDD have been found previously by some,^55, 56^ but not all studies.^57^ B2M was previously found to be increased in MDD compared to controls.^13^ Mesothelin is a glycoprotein that is highly expressed in certain cancers and is thought to have a role in cellular adhesion. No studies have previously linked mesothelin to depressive disorders.

### Metabolism and energy pathways

Adiponectin has a role in metabolic and immune processes and is negatively associated with obesity and insulin resistance.^58^ We found that adiponectin was lower in atypical depression versus controls. Previous studies have provided inconsistent findings with some studies finding decreased adiponectin levels in MDD^59, 60, 61, 62^ others found no difference,^58^ and one study reporting increased levels in MDD versus controls.^63^ One study found lower adiponectin levels in MDD cases with metabolic syndrome versus MDD cases without metabolic syndrome.^64^ This latter finding supports the suggestion that decreased adiponectin levels have a role in atypical depression only, as atypical depression has been found to be associated with metabolic syndrome.^49, 65^ Gluthathione *S*-transferase alpha 1 was not associated with MDD in a previous proteomic study;^12^ phosphoserine aminotransferase has not been investigated before, to our knowledge.

### Transport

Levels of SHBG can be decreased by high levels of insulin and are also decreased in diabetes.^66^ SHBG was not associated with depressive symptoms in a sample of persons with and without diabetes and insulin resistance.^66^

## Discussion

To our knowledge, this study is the first to identify serum proteins that are differentially expressed in atypical and melancholic depression subtypes versus controls using a proteomics approach. We observed differences in analytes between atypical depression and melancholic depression (9 analytes) and between atypical depression and controls (23 analytes), but not between melancholic depression and controls. There was overlap in the analytes identified in each of the three pairwise comparisons; of the 24 analytes, 8 (33%) emerged both in the comparison of atypical versus melancholic depression and in the comparison atypical depression versus control, indicating that these 8 markers could be specific to the atypical depressive subtype.

Many of the identified analytes are inflammatory markers or are involved in metabolism. This fits in with our previous findings of increased BMI, increased prevalence of metabolic syndrome and increased inflammation (CRP, interleukin-6 and tumor necrosis factor-α) in the atypical subtype,^22, 49^ and these findings support the idea that the atypical depressive subtype may in fact be a metabolic/inflammatory type of depression. In the past years, anti-inflammatory treatment has been increasingly investigated in trials with a recent meta-analysis showing a medium effect size in depression.^67^ However, this estimate was associated with considerable heterogeneity, and the authors recommended identification of subgroups that may benefit more from anti-inflammatory treatment. We hypothesize that the atypical subtype is such a subgroup.

Markers that may be of particular interest for future studies are the eight markers that seem specific to the atypical depressive subtype, namely leptin, IGFBP1 and IGFBP2, FABPa, insulin, mesothelin, C3 and B2M, all involved in either cell communication and signal transduction or immune responses. One other marker of interest is the ACE, which may be a marker that is specific to melancholic depression. As part of the renin–angiotensin system, ACE may be of interest because the renin–angiotensin system also partially regulates aldosterone secretion from the HPA-axis. Aldosterone and the renin–angiotensin system have been considered as potential biomarker and/or mediator in the pathophysiology of MDD.^51^

Although we controlled for various potential confounders, we did not control for BMI in the main analysis. It is known from the literature that there is a bidirectional relationship between BMI and depressive symptoms.^68^ This may be particularly the case for atypical depression as characterized by increased appetite and weight gain among other.^69, 70^ Atypical depression and increased BMI may also be a part of the same syndrome, as has been implied by the recent findings that atypical depression—but not melancholic depression—was associated with a variant of the obesity-related *FTO* gene and with a genomic profile risk scores for BMI.^71, 72^ Because of this evidence of (causal) associations between atypical depression and BMI, we consider BMI to be a mediator (rather than a confounder) and correction for BMI as an overcorrection. When we ran models for the 24 markers from Table 2 with additional correction for BMI to evaluate the mediating effects of BMI, results suggested mediation for most analytes (non-significant *P*-values and large changes in estimates). No mediation was found for IGFBP1, ACE and B2M (comparison atypical and melancholic depression) and B2M, ANG2 and VWF (comparison atypical depression and controls).

This paper follows a study evaluating changes in protein levels in all 6-month MDD cases and controls (*N*=1598) from the same cohort by Bot and colleagues. Only 5 of 33 markers previously found to be associated with MDD in the study by Bot *et al.*^11^ were also found to differ between controls and atypical depression in the present study (FABPa, ANG2, CathD, vWF and MIF), the observed directions of effect being consistent with the analysis by Bot *et al.* Notably, the markers in Bot *et al.* were derived before FDR correction and FDR adjustment showed that these markers were related to MDD only at a relatively high FDR level (range *q*-values 0.09–0.30). In contrast, in our study—with about half the number of MDD cases compared with the previous paper—we found that after FDR correction atypical depressed cases had significantly different levels of markers compared with controls and melancholic depression. Although differences in samples and analysis could explain some of the discrepancies, our results could be interpreted as proof of the usefulness of more homogenous subgroups of MDD for the identification of pathophysiological mechanisms. A *post hoc* comparison of estimates for the 171 analytes from the atypical depression versus controls model versus estimates from a model of all MDD cases (melancholic and atypical combined) versus controls showed a median fold-change of 1.45 for the estimates, demonstrating that associations get diluted when a more heterogeneous phenotype is used.

Strengths of this study include the relatively large sample size, with cases from the community, primary care and specialized care. Limitations include the fact that some analytes could not be evaluated because of levels below the level of detection. Also, the subtypes used were not based on stringent DSM-criteria, but are based on a data-driven analysis. However, the DSM definition of atypical depression has been criticized,^73, 74^ and other studies applying data-driven analysis of subtypes have yielded similar types^26, 27, 29, 30^ supporting the validity of these subtypes. Because assignment of persons to latent classes can be somewhat inaccurate for some persons,^11, 22^ we repeated analyses restricted to persons with a high classification accuracy (a posterior probability >0.80, that is, 65% of depressed cases). This showed highly comparable results, with overall stronger effects indicating that classification inaccuracy had little effect on overall conclusions. Furthermore, the decision to include markers with <30% missing values may be lenient and could have introduced error. However, using only markers with <20% missing values did not lead to different results, other than omission of two markers from Table 2 that had >20% missing values (INS and GSTa). Also, no proteomics replication samples were available. Although for some analytes we have found similar results using different assays (CRP^49^ and leptin ^40^) or have been found by others (CRP^18^ and leptin^39^), our proteomics results need to be replicated in independent samples. Unfortunately, independent replication of the identified changes is at present not possible for the following reasons: first, there is a lack of large cohorts and lack of detailed proteomic data for existing cohorts. Second, there are shortcomings in the assessment protocols for MDD in other studies; often, only change in appetite, weight and sleep are being recorded, rather than the direction of change. It would be recommendable to include direction of change in future data-collection protocols.

To conclude, we found no differences between the melancholic depressive subtype and controls, although the role of low levels of ACE in melancholic depression requires more research. We did find that the atypical depressive subtype had a differential protein profile compared to controls and the melancholic subtype. Identified proteins were mainly involved in cell communication and signal transduction, protein metabolism, immune response and metabolism and energy pathways, the latter two underlining the inflammatory and metabolic character of the atypical depressive subtype, and most of these associations seemed mediated by BMI. Although some uncertainties exist in our results, as a result of the missing data imputation and lack of proteomic replication samples, the presented results support the notion of atypical depression as a syndrome of metabolic disturbances that may benefit from add-on anti-inflammatory treatment. This study also suggests that depressive subtypes display distinct molecular profiles. Importantly, despite the need for additional validation studies, these results may provide the initial groundwork towards future patient stratification strategies that have more power to elucidate pathophysiological mechanisms of depression in research on biomarkers and pathophysiology.

## Figures and Tables

**Table 1:** Sample description (*N*=773)

	*Controls,* N=*414*	*Depressive cases*		*Overall* P-*value*
		*Severe melancholic subtype,* n=*231*	*Severe atypical subtype,* N=*128*	
Age, mean (s.d.)	39.0 (14.8)	41.7 (12.0)	40.7 (11.7)	0.05
Sex (% female)	60.6%	68.0%	71.9%	0.03
				
*Lab (%)*				*<0.001*
Amsterdam	15.7%	19.5%	12.5%	
Leiden	37.2%	49.8%	46.9%	
Groningen	43.2%	22.1%	29.7%	
Emmen	1.2%	7.8%	9.4%	
Heerenveen	2.6%	0.9%	1.6%	
				
Alcohol intake (p/week) median (IQR)	3.7 (1.0–8.7)	2.4 (0.2–8.7)	0.7 (0.0–4.2)	<0.001
Current smoker (%)	28.5%	50.6%	33.6%	<0.001
CVD (%)	2.9%	10.4%	5.5%	<0.001
Diabetes (%)	3.9%	8.7%	6.3%	0.04
IDS severity score, mean (s.d.)	8.0 (7.1)	38.6 (9.7)	39.1 (8.8)	<0.001
Comorbid anxiety, *N* (%)	NA	142 (61.5%)	83 (64.8%)	0.53
SSRI, *N* (%)	3 (0.7%)	78 (33.8%)	47 (36.7%)	<0.0001
TCA, *N* (%)	0 (0%)	10 (4.3%)	3 (2.3%)	<0.001
Other AD, *N* (%)	0 (0%)	32 (13.9)	10 (7.8%)	<0.001
Anti-inflammatory agents use, *N* (%)	4 (1.0%)	16 (6.9%)	9 (7.0%)	<0.0001
Corticosteroid use, *N* (%)	15 (3.6%)	17 (7.4%)	12 (9.4%)	0.02
BMI, mean (s.d.)	24.8 (4.6)	25.3 (5.2)	28.7 (6.3)	<0.0001

Abbreviations: AD, antidepressants; BMI, body mass index; CVD, cardiovascular disease; IDS, Inventory of Depressive Symptomatology; IQR, interquartile range; NA, not applicable; SSRI, selective serotonin reuptake inhibitors; TCA, tricyclic antidepressants.

**Table 2:** Overview of biomarkers significantly^a^ differing between depressive subtypes and controls

*Analyte*	*Biological process (HPRD)*	*Atypical subtype vs melancholic subtype (ref)*				*Atypical subtype vs control (ref)*				*Melancholic subtype vs control (ref)*			
		b	*s.e.*	P-*value*	q*-value*	b	*s.e.*	P*-value*	*q-value*	b	*s.e.*	P-*value*	q*-value*
MSLN	CA, IR	−0.070	0.022	0.001	0.026	−0.058	0.020	0.004	0.034	0.012	0.017	0.483	0.898
Leptin	CC, ST	0.227	0.045	5.426E−07	9.278E−05	0.251	0.042	2.588E−09	4.426E−07	0.024	0.035	0.502	0.898
IGFBP1	CC, ST	−0.256	0.053	1.398E−06	1.195E−04	−0.194	0.049	7.296E−05	2.495E−03	0.062	0.041	0.135	0.601
IGFBP2	CC, ST	−0.094	0.023	3.246E−05	0.001	−0.072	0.021	0.001	0.010	0.023	0.018	0.198	0.730
FABPA	CC, ST	0.084	0.023	2.927E−04	0.010	0.101	0.021	3.308E−06	2.828E−04	0.016	0.018	0.373	0.861
INS	CC, ST	0.129	0.038	0.001	0.021	0.138	0.036	1.149E−04	0.003	0.009	0.030	0.758	0.996
C3	IR	0.037	0.010	3.780E−04	0.011	0.042	0.010	1.319E−05	0.001	0.005	0.008	0.533	0.898
B2M	IR	0.039	0.012	0.001	0.023	0.047	0.011	2.926E−05	0.001	0.007	0.009	0.439	0.898
ACE	PM	0.059	0.014	2.737E−05	0.001	0.023	0.013	0.074	0.247	−0.036	0.011	0.001	0.192
													
ANG2	CC, ST	−0.017	0.021	0.429	0.649	−0.066	0.020	0.001	0.010	−0.050	0.017	0.003	0.242
CPep	CC, ST	0.068	0.024	0.005	0.068	0.066	0.022	0.003	0.027	−0.002	0.019	0.931	0.999
MIF	CC, ST	0.038	0.029	0.188	0.460	0.078	0.027	0.004	0.032	0.040	0.023	0.077	0.533
GH	CC, ST	−0.066	0.059	0.264	0.557	−0.157	0.055	0.004	0.034	−0.091	0.046	0.051	0.459
A1Micr	IR	0.024	0.010	0.013	0.127	0.033	0.009	2.680E−04	0.006	0.009	0.008	0.257	0.757
CRP	IR	0.169	0.061	0.006	0.069	0.175	0.056	0.002	0.020	0.006	0.048	0.902	0.999
PSAT	M, EP	0.073	0.026	0.005	0.068	0.089	0.024	2.266E−04	0.006	0.016	0.020	0.443	0.898
GSTa	M, EP	0.119	0.043	0.006	0.069	0.127	0.040	0.001	0.018	0.008	0.034	0.821	0.996
APN	M, EP	−0.051	0.023	0.028	0.186	−0.062	0.022	0.004	0.034	−0.010	0.018	0.569	0.926
SAP	PM	0.034	0.015	0.020	0.155	0.047	0.014	4.767E−04	0.009	0.013	0.011	0.244	0.731
TPA	PM	0.040	0.018	0.024	0.175	0.055	0.016	0.001	0.010	0.015	0.014	0.277	0.777
CathD	PM	0.022	0.012	0.073	0.271	0.035	0.011	0.002	0.020	0.013	0.010	0.171	0.712
VWF	PM	0.034	0.020	0.087	0.298	0.057	0.018	0.002	0.020	0.023	0.015	0.131	0.601
AACT	PM	0.011	0.012	0.339	0.594	0.030	0.011	0.006	0.042	0.019	0.009	0.040	0.446
SHBG	T	−0.081	0.028	0.004	0.068	−0.088	0.026	0.001	0.010	−0.007	0.022	0.746	0.996

Abbreviations: A1Micr, alpha-1-microglobulin; AACT, alpha-1-antichymotrypsin; ACE, angiotensin-converting enzyme; ANG2, angiopoetin-2; APN, adiponectin; *b*, regression coefficient; B2M, beta-2-microglobulin; C3, Complement C3; CathD, Cathepsin D; CA, cell adhesion; CC, cell communication; Cpep, C-peptide; CRP, C-reactive protein; CVD, cardiovascular disease; EP, energy pathways; FABPA, fatty-acid-binding protein, adipocyte; FDR, false discovery rate; GH, human growth hormone; GSTa, glutathione *S*-transferase alpha; HPRD, Human Protein Reference Database; IGFBP1, insulin-like growth factor-binding protein 1; IGFBP2, insulin-like growth factor-binding protein 2; INS, insulin; IR, immune response; M, metabolism; MIF, macrophage migration inhibitory factor; MSLN, mesothelin; PM, protein metabolism; PSAT, phosphoserine aminotransferase; SAP, serum amyloid P-component; SHBG, sex hormone-binding globulin; ST, signal transduction; T, transport; TPA, tissue-type plasminogen activator; VWF, von Willebrand factor.

a On the basis of *q*-value (FDR corrected). Model corrected for age, sex, lab, smoking, alcohol intake, CVD and DM.
