# Supplementary material for: Serum proteomic profiles of depressive subtypes
Source: Transl Psychiatry. 2016 Jul 12;6(7):e851–. doi: 10.1038/tp.2016.115 (PMC5545705; doi:10.1038/tp.2016.115)
Supplement: Supplementary Figure 1 [file tp2016115x1.pdf]

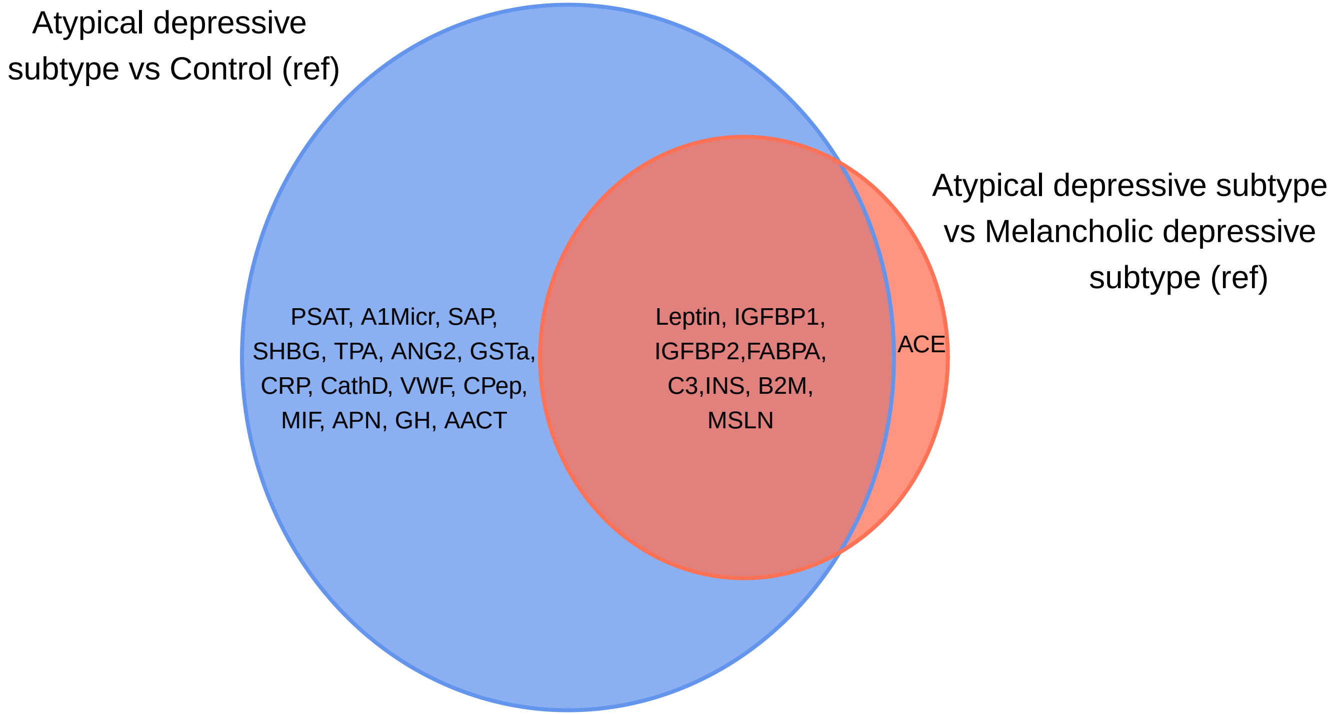
Atypical depressive subtype vs Control (ref)
Atypical depressive subtype vs Melancholic depressive subtype (ref)
PSAT, A1Micr, SAP, SHBG, TPA, ANG2, GSTa, CRP, CathD, VWF, CPep, MIF, APN, GH, AACT
Leptin, IGFBP1, IGFBP2,FABPA, C3,INS, B2M, MSLN
ACE
